# A complete mitochondrial DNA genome of a mangrove clam *Geloina expansa* (Mousson, 1849)

**DOI:** 10.1080/23802359.2026.2715807

**Published:** 2026-08-18

**Authors:** Xuan-Yu Chen, Zhan-Wei Zhao, Yun-Wei Dong

**Affiliations:** ^a^ Key Laboratory of Mariculture of Ministry of Education, College of Fisheries, Ocean University of China, Qingdao, China; ^b^ Shandong Key Laboratory of Green Mariculture and Smart Fishery, Fisheries College, Ocean University of China, Qingdao, China; ^c^ Function Laboratory for Marine Fisheries Science and Food Production Processes, Qingdao Marine Science and Technology Center, Qingdao, China

**Keywords:** *Geloina expansa*, mangrove clam, mitochondrial genome rearrangement, phylogenetic analysis

## Abstract

The mangrove clam (*Geloina expansa*) is widely distributed in Asia, but its phylogenetic relationship to congeners (especially *G. erosa*) remains unclear. The complete mitochondrial genome of *G. expansa* was 15,187 bp and contained the typical 37 genes. Most protein-coding genes started with ATN and terminated with TAA or TAG. Some genes exhibited a different order from those in published *Geloina* mitogenomes, suggesting gene rearrangement. Phylogenetic analysis revealed that *G. expansa* clustered with one *G. erosa* mitogenome. Their small genetic distance (0.029) supports conspecificity, whereas another *G. erosa* individual showed a distance of 0.189, suggesting an unidentified species within *Geloina*.

## Introduction

*Geloina*, a typical bivalve genus in mangrove ecosystems, is widely distributed along the coasts of India, Vietnam, Malaysia, and southeastern China. It possesses both significant ecological and economic value and holds great potential as an indicator for marine monitoring as well as a sustainable, high-nutrient-density food source (Morton 1976; Cai et al. 1995; Clemente and Ingole 2009; Wang et al. 2020; Pai et al. 2025). However, because species in the genus *Geloina* occupy similar habitats and exhibit similar appearances, taxonomic methods have proven inadequate for distinguishing among *Geloina* species based on shell shape alone (Nong et al. 2015).

Previous studies treated *G. erosa* and *G. expansa* as two distinct species (Liao et al. 2020; Wu et al. 2023). However, *G. erosa* is no longer recognized as a valid species and is considered a synonym of *Geloina expansa* in the World Register of Marine Species (WoRMS). To address this controversial issue, we sequenced the complete mitochondrial genome (mitogenome) of *G. expansa* for the first time and conducted a phylogenetic analysis to clarify its relationships and taxonomic status within the genus *Geloina*. This research aims to provide a more accurate evaluation of the genus’s phylogenetic structure.

## Material and methods

### Material collection, DNA extraction, and sequencing

**Figure 1.**
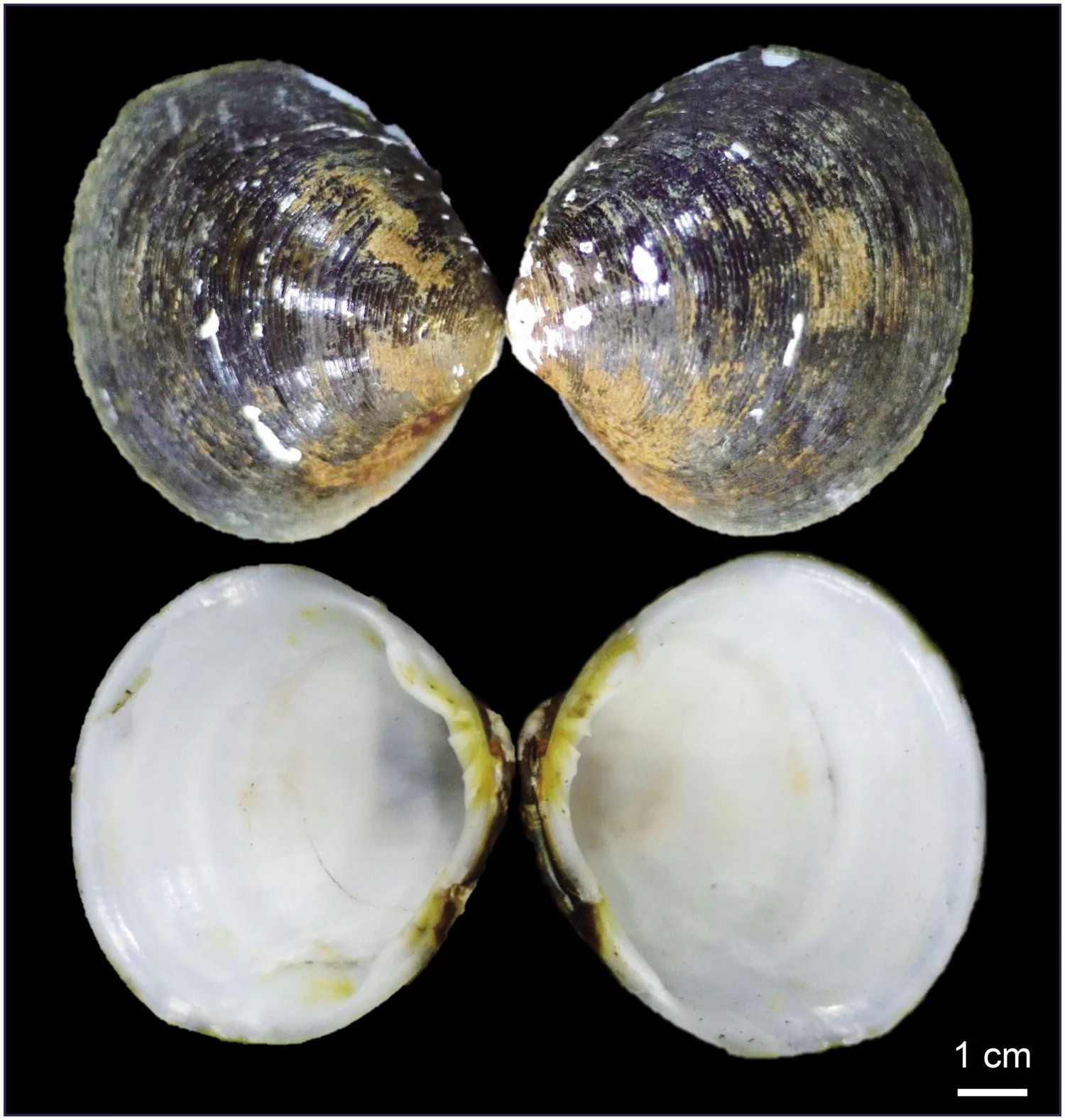
Shell morphology of *Geloina expansa* (Mousson, 1849) (voucher: LInE-Gex001), collected from muddy sediment in a mangrove habitat in Beihai City, Guangxi Province, China. External views (upper row) and internal views (lower row) of the left and right valves. The shell is thick, equivalve, strongly inflated, and trigonal-ovate in outline, with prominent umbones. The external surface is covered by a dark brown to black periostracum and bears distinct concentric growth lines. The internal surface is smooth and white, with visible adductor muscle scars; the ventral margin is smooth. The illustrated specimen has a shell length of 65.02 mm, shell height of 63.25 mm, shell width of 37.31 mm, and a ligament width of 4.41 mm. Scale bar = 1 cm. Photograph by Xuan-Yu Chen.

Specimens of *G. expansa* (Figure 1) were collected in the mangrove located at Beihai City, Guangxi Province, China (21.54°N, 109.76°E). A specimen is deposited at the Laboratory of Intertidal Ecophysiology, Fisheries College, Ocean University of China (Zhan-Wei Zhao, zhaozhanwei@stu.ouc.edu.cn) under the voucher number LInE-Gex001.

The specimen was initially identified as *Geloina expansa* based on shell morphology and mangrove habitat information. Because *Geloina* species are morphologically similar and COI sequences do not clearly separate *G. expansa* from *G. erosa* (Cai et al. 1995; Wu et al. 2023), we treated the specimen as *G. expansa* under the current taxonomic treatment, while acknowledging the unresolved boundary within the *G. expansa*/*G. erosa* complex.

Total genomic DNA was extracted using the CTAB method (Ding et al. 2018). The extracted DNA sample was sent to Beijing Novogene Co., Ltd., where sequencing was performed on the Illumina NovaSeq 6000 platform, yielding 150-bp paired-end reads. Raw sequencing reads were quality-filtered using fastp v0.23.2 (Chen et al. 2018), resulting in 276,135,472 paired-end read pairs for downstream analyses.

### Mitogenome assembly and annotation

The mitogenome was assembled using NOVOPlasty v4.3.5 (Dierckxsens et al. 2017) with the mitogenome of Villorita cyprinoides (NC_050989) as the seed reference and K-mer values of 23, 33, and 39. Assembly reliability was evaluated using MitoFinder v1.4.2 (Allio et al. 2020) and MEANGS v3 (Song et al. 2022). Sequencing depth was calculated with GetOrganelle v1.7.7.1 (Jin et al. 2020). Gene annotation was performed using MITOS (Bernt et al. 2013) with the invertebrate genetic code and RefSeq89 Metazoa reference dataset, while all other parameters were kept at default settings. Finally, the circular map of the mitogenome was generated using the online server CGView (https://proksee.ca/) (Grant and Stothard 2008).

### Phylogenetic tree construction

The Bayesian inference (BI) phylogenetic tree was constructed using the plug-in MrBayes program in PhyloSuite v.1.2.3 (Zhang et al. 2020) based on nucleotide sequences of 13 PCGs and 2 rRNA genes of 10 mitogenome sequences retrieved from NCBI GenBank (https://www.ncbi.nlm.nih.gov/genbank/) (see Figure 3 legend for details). To evaluate the phylogenetic position of *G. expansa* within the genus *Geloina*, all available complete mitochondrial genomes of genus *Geloina* were included in the analysis. Representative mitogenomes from other members of Cyrenoididae were additionally incorporated to provide broader phylogenetic context. *Ruditapes decussatus* (NC_084339) and *Mercenaria mercenaria* (NC_048487) were included as outgroups. The Bayesian analysis involved four independent runs of 300,000 generations of Markov Chain Monte Carlo (MCMC), with four chains per run. Sampling was conducted every 1,000 generations, and the first 25% of samples were discarded as burn-in. Convergence and mixing of the MCMC chains were assessed using Tracer v1.7.2 (Rambaut et al. 2018). The best-fitting substitution model and partitioning schemes were selected using Modelfinder v1.4.2 (Kalyaanamoorthy et al. 2017). The BI tree was viewed and edited using FigTree v1.4.4 (http://tree.bio.ed.ac.uk/software/figtree/). We constructed the Maximum Likelihood (ML) phylogenetic tree and calculated genetic distances using the concatenated sequence of 13 PCGs and 2 rRNAs under the Kimura-2-parameter (K2P) model, with 2000 bootstrap replicates, in MEGA 12 (Kumar et al. 2024).

## Results

**Figure 2.**
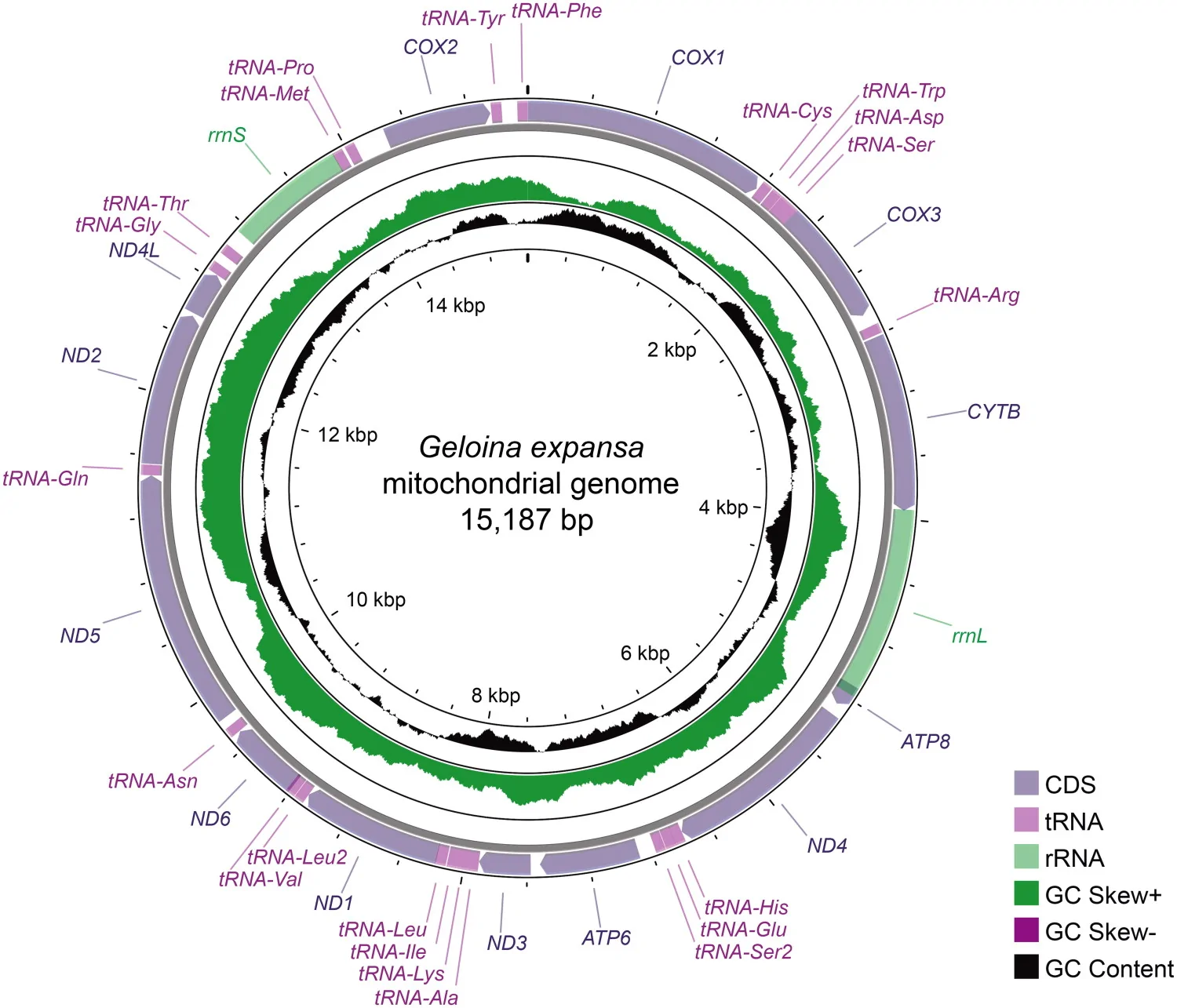
Complete mitogenome circular map of *Geloina expansa*, blue represents PCGs, purple represents tRNA, green represents rRNA, black areas represent GC content; in addition, the map also shows the GC-skew value.

The length of the complete mitogenome of *G. expansa* was 15,187 bp (GenBank accession no. PZ255822), with an average depth of coverage of 9,264× (Figure S1). It owned 13 PCGs, 2 rRNA genes and 22 tRNA genes (Figure 2). The A + T content of *G. expansa* was 68.77%. Except for *cytb* (start with TTG), 12 PCGs started with an ATN (ATA, ATG and ATT) initiation codon. Except for *cox3*, which possessed an incomplete termination codon (TA-), 12 PCGs were terminated with TAG or TAA stop codons. All 13 PCGs were encoded on the heavy strand (Table 1).

**Table 1.** PCG composition of the complete mitogenome of *Geloina expansa*, including length, strand orientation, and start codon and stop codons.

PCG	Length	Strand	Start codon	Stop codon
*cox1*	1578	+	ATT	TAA
*cox2*	687	+	ATT	TAA
*cox3*	785	+	ATA	TA-
*atp6*	636	+	ATG	TAG
*atp8*	153	+	ATG	TAG
*nad1*	915	+	ATA	TAG
*nad2*	948	+	ATG	TAG
*nad3*	333	+	ATT	TAA
*nad4*	1236	+	ATG	TAA
*nad4l*	285	+	ATG	TAA
*nad5*	1650	+	ATT	TAA
*nad6*	486	+	ATG	TAG
*cytb*	1134	+	TTG	TAG

**Figure 3.**
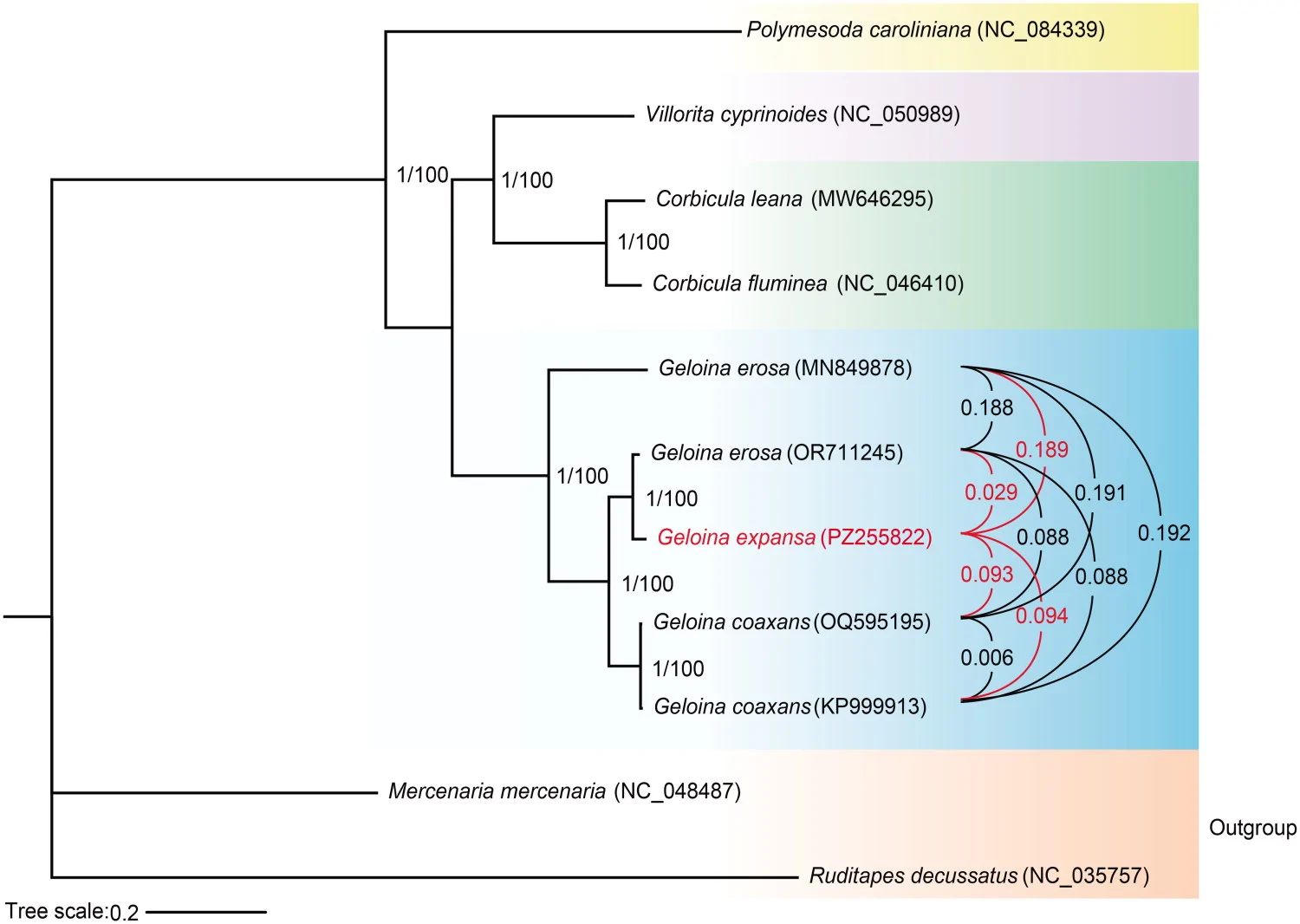
Bayesian inference (BI) and maximum likelihood (ML) phylogenetic trees based on 13 PCGs and 2 rRNA sequences. Posterior probabilities above 0.7 (BI) and bootstrap support values above 70% (ML) are shown. The new sequence in this study is marked in red. The following sequences were used: NC_084339 (Kwak et al. 2024), NC_050989 (Rahuman et al. 2020), MW646295, NC_046410 (Zhang et al. 2019), MN849878 (Liao et al. 2020), OR711245 (Kwak et al. 2024), OQ595195 (Wu et al. 2023), KP999913, NC_048487 (Hu et al. 2019) and NC_035757.

The best-fit model selected by ModelFinder based on BIC was GTR+I + G. Convergence was adequate, with all effective sample size (ESS) values >200 and the average standard deviation of split frequencies (ASDSF) of 0.000 across independent runs. The phylogenetic tree indicated that *G. expansa* and one *G. erosa* individual (OR711245) clustered together, followed by *G. coaxans*, and then another *G. erosa* (MN849878) (Figure 3). All genetic distances mentioned hereafter were calculated based on the concatenated alignment of 13 PCGs and 2 rRNA genes (units: substitutions per site). COI-based distances are shown in parentheses for comparison.

Pairwise K2P genetic distances among the analyzed mitogenome sequences ranged from 0.006 (COI: 0.004) to 0.192 (COI: 0.123). The distance between *G. erosa* (OR711245) and *G. expansa* (PZ255822) was 0.029 (COI: 0.024), which was lower than the distances between *G. erosa* and *G. coaxans*, but higher than the observed intraspecific distance within *G. coaxans* [0.006 (COI: 0.004)]. In contrast, the distance between another sequence annotated as *G. erosa* (MN849878) and *G. expansa* (PZ255822) was much higher [0.189 (COI: 0.119)]. These contrasting patterns suggest that sequences currently annotated as *G. erosa* in public databases are genetically heterogeneous. Therefore, the available genetic-distance data do not provide a clear threshold for distinguishing intra- and interspecific divergence within the *G. expansa*/*G. erosa* complex, and should be interpreted cautiously.

**Figure 4.**
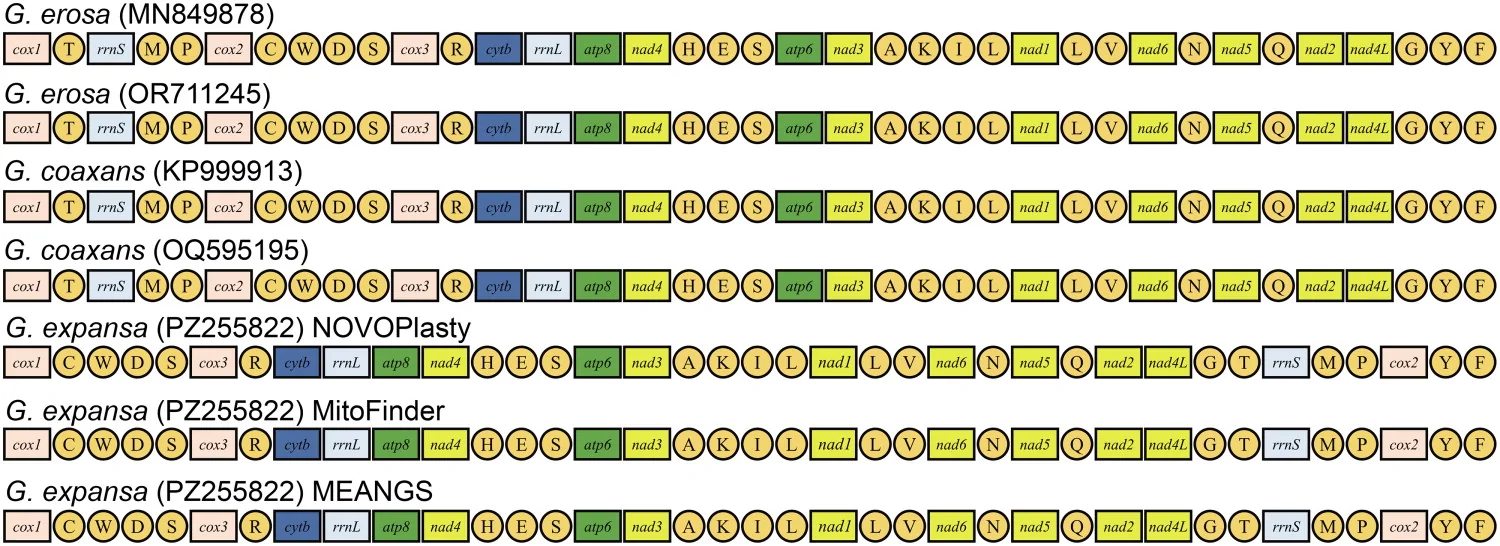
The gene arrangements of different samples of *Geloina*. The mitogenome of *Geloina expansa*, assembled using NOVOPlasty, MitoFinder, and MEANGS respectively, consistently exhibited a distinct genomic arrangement compared to other species within the genus *Geloina.*

The mitogenome of *G. expansa* showed a different gene order compared with other mitogenomes of other *Geloina* species, and the mitogenome of the *G. expansa* individual exhibits an excision of the region spanning from tRNA-Thr to *cox2*, which is subsequently inserted between tRNA-Gly and tRNA-Tyr (Figure 4).

## Discussion and conclusion

This study provides a complete mitogenome for *Geloina expansa*, offering valuable data for resolving taxonomic and phylogenetic questions within the genus. Phylogenetic analyses based on linked mitochondrial genes reveal a close relationship between *G. expansa* (PZ255822) and an individual of *G. erosa* (OR711245), supporting recent taxonomic updates that classify *G. erosa* as a synonym of *G. expansa*. However, the noticeable genetic difference seen in another *G. erosa* individual (MN849878) suggests the existence of cryptic species within the genus, indicating that current classifications should be further examined.

Notably, the gene order of the newly assembled mitogenome of *G. expansa* differs from that of all previously known species within the genus *Geloina*. The discovery of mitochondrial gene rearrangement in *G. expansa*, involving rRNA, tRNAs and protein-coding genes, this is consistent with the well-established finding that mitochondrial gene order in Bivalvia is highly rearranged (He et al. 2011; Plazzi et al. 2016; Rahuman et al. 2020). The consistency of this pattern across multiple assembly strategies (NOVOPlasty, MitoFinder and MEANGS) supports its authenticity and suggests that mitogenome architecture in bivalves may be more dynamic than previously recognized.

In summary, this study provides new mitogenomic data that help evaluate the phylogenetic placement of *G. expansa* and the evolutionary relationships within *Geloina*. Further studies with broader sampling and additional genomic markers are needed to clarify species boundaries and mitogenome evolution in this genus.

## Supplementary Material

Supplementary.docx

## Data Availability

The genome sequence data that support the findings of this study are openly available in GenBank of NCBI at https://www.ncbi.nlm.nih.gov/under the accession no. PZ255822. The associated BioProject, SRA, and Bio-Sample numbers are PRJNA1450030, SRR37990761, and SAMN57140883, respectively.
