# Supplementary material for: A complete mitochondrial DNA genome of a mangrove clam Geloina expansa (Mousson, 1849)
Source: Mitochondrial DNA B Resour. 2026 Aug 18;11(9):1048–53. doi: 10.1080/23802359.2026.2715807 (PMC13487844; doi:10.1080/23802359.2026.2715807)
Supplement: Supplementary file 1 [file TMDN_A_2715807_SM5368.docx]

**A complete mitochondrial DNA genome of a mangrove clam *Geloina expansa* (Mousson, 1849)**

**Figure S1.** Analysis of sequencing coverage depth for the mitogenome of G. expansa using GetOrganelle v1.7.7.1. The green, blue, yellow, and red lines in the figure represent the coverage depth of matched, mismatched, inserted, and deleted bases, respectively.
